# Dietary Approaches from Moms, Farms, and Nature to Overcome Chronic Diseases and the Pharmacracy

**DOI:** 10.3390/nu15183965

**Published:** 2023-09-14

**Authors:** Rodney R. Dietert

**Affiliations:** Department of Microbiology and Immunology, Cornell University, Ithaca, NY 14853, USA; rrd1@cornell.edu

Chronic diseases, previously called noncommunicable diseases, are the leading cause of global death and were recently estimated by the World Health Organization to account for 74% of all deaths [1]. These diseases, such as Alzheimer’s Disease, autism, cardiovascular diseases, obesity, diabetes, stroke, hypertension, chronic kidney disease, autoimmune conditions, and cancer, not only kill but also severely reduce the human health span (years spent in wellness) [2,3]. They rarely occur in isolation as we age and instead propagate in numbers via a spider web of interlinked and predictable multi-morbidities (two or more co-existing chronic diseases) [4,5]. These diseases require ever-increasing medical and prescription drug interventions as we age [6,7] and can be devastating for families in terms of caregiver impact and needs [8]. We must learn from the past to devise a healthier future, and dietary approaches to chronic diseases, as covered in this Special Issue, is a key part of the solution. Other researchers have reached a similar conclusion [9,10,11].

We have experienced a half-century of pharma-driven medical strategies as humanity’s solution against chronic diseases. But this pharmacratic-driven way of life is coming to an end. The public was promised cures but, instead, received multimorbidity and polypharmacy. An avalanche of new drugs over the past 50 years produced a dizzying array of drug incompatibilities and “side effects”, most of which have developed into additional chronic diseases, each with their own medically-coded diagnoses and new prescription drugs. The cycle is just as never-ending as chronic disease epidemics are unrelenting.

Our prescription drug-filled lifespan features a higher multimorbidity burden, increased polypharmacy, and record deaths by chronic diseases. While disease symptoms are often dampened, the underlying causes and dysfunctions usually persist only to insidiously promote additional diseases (e.g., cancers developing in tissue targets of earlier-life chronic diseases) [12,13]. Of course, the reverse is also true, where many adults who survive cancer only find that they carry an increased risk and burden of additional chronic diseases [14]. As examples of the current comorbidity problem, two diseases that often emerge during childhood are asthma and obesity, which can be seen as entryway diseases and eventual multimorbidity [15]. Recently, asthma was found to present an elevated risk for 36 additional comorbid chronic diseases, while obesity has an elevated risk for 43 conditions [16]. Mis-regulated inflammation is at the center of most chronic diseases [17,18]. There is a clear pattern in which a chronic disease diagnosis leads to drug treatment followed by a new comorbid chronic disease diagnosis and more specific drug treatments. This is how we find ourselves in a state of multimorbidity and polypharmacy. The additional comorbidities are quite predictable, and Mendito et al. recently characterized several well-described disease propagation patterns [19]. The sad reality is that we have witnessed the emergence of a new national pastime: pill-popping.

Enter a new era driven by dietary approaches in which moms, farms, and nature rather than pharma are the foundational sources of chronic disease prevention and cures. Of course, this new era has its roots in earlier days of human history when our daily lives were immersed in both food collection/production and nature. Elevating the use of dietary approaches for increased prevention and treatment of chronic diseases offers an attractive alternative to what can only be characterized as a failed half-century in the fight to reduce our burden of chronic disease. As a strategy, dietary approaches cover a massive area of intervention opportunities ranging in scope from the self-health empowerment of individuals and families to refocused public health policies. Dietary approaches can include not only macronutrients and micronutrients but also factors such as nutraceuticals, supplements, fermented foods, probiotics, prebiotics, synbiotics, herbs, plant extracts/medicinals, fasting, circadian rhythm integration/eating patterns, as well as food source variables (production/storage/preparation), organic and GMO- and antibiotic-free production, different crop production methods, phytochemical loading in animal production (e.g., regenerative agriculture), etc. One of the already obvious considerations is that “healthy” eating may benefit large populations. However, it is not surprising that one diet does not fit all. We came from different ancestors living in different regions of the earth with different climates, on different soils, with different diets, possessing significantly different microbiomes, and consuming different fermented foods. Dietary approaches should include recognition of the need for personalization.

Three examples are introduced to emphasize the power of dietary approaches to reduce the burden of chronic diseases and polypharmacy (one from each of the categories: moms, farms, and nature). Dietary conditions in early life can greatly affect the risk of chronic diseases. Among the initial observations of this relationship was that of Dr. David Barker, MD, who described the relationship between fetal nutrition and later-life cardiovascular disease [20]. We now know that critical windows of vulnerability exist relative to chronic disease risk. Among those is a critical window in early neonatal development in which specific Bifidobacteria (e.g., *Bifidobacterium longum* ssp. *infantis)* must seed the infant gut, and those bacteria must be fed with human milk oligosaccharides (HMOs) in breast milk to mature the infant immune system and greatly reduce the risk of allergic diseases, including asthma [21,22]. Moms naturally direct this process via natural childbirth and breastfeeding when possible. However, problems can arise with natural childbirth and/or breastfeeding, and it was recently shown that dietary and probiotic intervention can assist if needed [23,24]. The significance of this diet and microbiome-related window was recently described by Hoskinson et al. [25], where diet-driven specific microbiota maturation must occur to prevent an early childhood entryway chronic disease.

A second example relates to farms and food production. Farm living in the absence of pesticides can be a risk reduction factor for many chronic diseases, and at least some of this protection appears to be related to specific farm-associated diets. For example, raw milk consumption has been identified in meta-analyses and reviews as a beneficial factor to protect against asthma and allergies in children [26,27]. A recent raw cow’s milk benefit-risk evidence map was described by Dietert et al. [28].

In this case, a third example of dietary approaches stemming from nature concerns curcumin. The polyphenol curcumin (diferuloylmethane) is a polyphenol extracted from the plant turmeric (Curcuma longa). It is widely used as a spice component or coloring agent and has been a staple in both traditional Chinese and traditional Indian (e.g., Ayurveda) medicine. It is a potent anti-inflammatory agent, and for this reason, it has many applications in preventing and treating inflammation-driven chronic diseases. Examples include cardiovascular diseases [29], metabolic diseases [30], and neurological diseases [31]. This anti-inflammatory agent is so useful against chronic diseases that at least one Special Issue among MDPI journals has been devoted to this single spice [32]. Sadeghi et al. [33] recently described the broader range of curcumin properties (anti-inflammatory, immune-regulatory, anti-oxidative, and lipid-modifying properties) in characterizing its mechanism of action against inflammatory diseases.

These three examples illustrate the breadth of opportunities for dietary approaches to combat the ongoing chronic disease epidemic. This Special Issue provides a forum to (1) showcase dietary approaches to prevent and/or treat chronic diseases and (2) help to end myopic organ/tissue-based pharmaceutical approaches that have failed to adequately address the underlying causes of chronic diseases and have propagated polypharmacy. Diet ultimately drives the microbiome for the human holobiont, which, in turn, drives the development and function of our body’s integrated systems. This is a route through which we can extend the human healthspan.
